# Adipose Tissue, at the Core of the Action of Incretin and Glucagon-Based Anti-Obesity Drugs

**DOI:** 10.1007/s13679-025-00660-w

**Published:** 2025-09-02

**Authors:** Francesc Villarroya, Marion Peyrou, Marta Giralt

**Affiliations:** 1 Departament de Bioquímica i Biomedicina Molecular, Departament de Bioquímica i Biomedicina Molecular, Facultat de Biologia, Universitat de Barcelona, Avda Diagonal 643, Barcelona, Catalonia 08028 Spain; 2 Institut de Biomedicina de la Universitat de Barcelona (IBUB), Barcelona, Spain; 3 Institut de Recerca de Sant Joan de Déu, 08028 Barcelona, Spain; 4 CIBER Fisiopatología de la Obesidad y Nutrición (CIBEROBN), Madrid, 28029 Spain

**Keywords:** Glucagon-like peptide-1, Glucose-dependent insulinotropic peptide, Glucagon, White adipose tissue, Brown adipose tissue, Obesity

## Abstract

**Purpose of the Review:**

This review summarizes recent evidence highlighting the specific role of adipose tissue in the systemic effects of incretin agonist-based drugs used in the treatment of obesity.

**Recent Findings:**

The development of incretin agonist-based drugs has achieved unprecedented success in the pharmacological treatment of obesity and the improvement of obesity-related comorbidities. While initially shown to significantly reduce adipose tissue through decreased food intake, incretin-based therapy is also increasingly reported to alter the properties of adipose tissue. Recent experimental and human studies indicate that these anti-obesity drugs induce significant changes in the metabolism and inflammatory state of adipose tissue, while also promoting its thermogenic plasticity.

**Summary:**

The direct and indirect actions of incretin-based anti-obesity drugs, which modify the properties of adipose tissue, are emerging as key contributors to the systemic health benefits of these treatments.

## Introduction

Obesity has become a widespread global issue and 1 in 8 people worldwide were living with obesity in 2022 [1]. The recommended lifestyle changes alone have very moderate weight-loss effects in people living with obesity, and bariatric surgery is currently considered the most effective treatment for inducing weight loss which, in addition to promoting weight loss, improves type 2 diabetes (T2D) and reduces various cardiometabolic risk factors [2]. Bariatric surgery is regarded as relatively safe, but this invasive procedure can have significant side effects, and there is considerable variability in long-term outcomes among patients.

Researchers have long sought to develop effective non-surgical pharmacological strategies to treat obesity. Historically, these efforts met limited success, having only modest effects on body weight and/or triggering harmful side effects. In recent years, the emergence of drugs that are based on incretins (a group of gut hormones increasing insulin secretion and stimulating a decay in blood glucose levels), such as those acting as glucagon-like peptide-1 (GLP-1) and glucose-dependent insulinotropic peptide (GIP) agonists, has marked an unprecedented success in the treatment of obesity. Millions of people are now being treated with incretin agonism-based anti-obesity medications, making it a global priority to fully understand their action mechanisms and systemic effects and further enhance their safety and efficacy. Samms and Kusminski have recently provided an up-to-date, comprehensive overview of the current understanding of the mechanisms of action of incretin-based drugs and their significance in obesity research [3].

GLP-1 is an incretin that is produced and secreted primarily by intestinal L cells, as well as by certain neurons and a subset of pancreatic α cells in the islets of Langerhans [4, 5]. It acts on target tissues through interaction with the GLP-1 receptor (GLP-1R). GLP-1 helps to lower blood sugar by stimulating insulin secretion and inhibiting glucagon release. In the brain, GLP-1 reduces appetite; in the stomach, it slows gastric emptying; in insulin-sensitive tissues, such as muscle and adipose tissue, it enhances glucose uptake [6]; in the liver, it inhibits glucose production [7]. Additionally, GLP-1 has demonstrated cardioprotective effects [7].

GIP is an incretin produced and secreted by intestinal K cells. Its receptor, GIPR, is found in pancreatic β-cells as well as in many other tissues and organs. Like GLP-1, GIP is an incretin, so it lowers blood sugar by enhancing pancreatic insulin secretion [8]. Additionally, GIP has been shown to act on other tissues, particularly affecting lipid metabolism and cardiovascular system [9, 10].

Both GLP1-R and GIPR are members of the G-protein coupled receptor family and, upon activation, induce adenylate cyclase activity and increase intracellular cAMP, which acts as a key intracellular mediator of their biological actions [11].

Glucagon (GCG) is a peptide hormone produced and secreted by α cells in the pancreas, mostly in response to fasting stimulus [12]. GCG primarily functions to prevent glucose levels from falling too low by stimulating glucose production from the liver and regulating hepatic fatty acid metabolism. For its biological actions, glucagon acts on the glucagon receptor (GCGR), also a G-protein coupled receptor increasing cAMP when activated. GCGR is present preferentially in the liver but also in extra-hepatic cells and organs such as islet β cells, brain, adipose tissue, heart, and kidney [13]. Given the hyperglycaemic effect of GCG, it might seem counterintuitive to use GCG agonism in people living with obesity. However, it is well-known to induce satiety, slow emptying of the stomach and enhance energy expenditure [14–16]. Thus, in recent years it has been proposed that combining GCG agonism with incretin-based agonism could help optimize the pharmacological treatment of obesity.

Body weight regulation is mainly governed by the brain and adipose tissue. The highly plastic adipose tissue serves multiple functions, ranging from the classical role of lipid storage in white adipose tissue (WAT) to the dissipation of energy through thermogenic processes, such as in brown adipose tissue (BAT). In obesity, the enlargement of WAT is associated with hypertrophy and, in some cases, hyperplasia of adipocytes, which enable WAT to increase fat storage in response to a positive energy balance [17]. The brain and adipose tissue communicate via signalling networks, such as those mediated by adipose tissue-released adipokines, including leptin, adiponectin and many others. Adipokines act centrally and peripherally to regulate energy homeostasis and the metabolic response to changes in fat mass [18]. Incretin agonism-based treatments, in association with their strong weight-reducing effects, significantly impact adipose tissue through both direct and indirect mechanisms, and they are likely to affect not only the quantity of adipose tissue but also its specific anatomical and cellular characteristics, and thus could have profound implications for patients’ health.

## GLP-1R Agonism and White Adipose Tissue

### GLP-1R Agonist Treatments and their Effects on Body Weight and Fat Mass

Given the biological properties of GLP-1, researchers had long envisioned the potential of its therapeutic use. However, native GLP-1 proved to be of limited use as a drug candidate, because it exhibits rapid proteolytic degradation and renal clearance, and thus has a very short half-life. Peptidic GLP-1-based drugs were developed by introducing amino acid sequence changes and chemical additions that can prevent inactivation and favour stability without altering the agonist properties. Given the incretin effects of GLP-1, GLP-1R agonists were initially developed to treat T2D. However, it was clear from the initial clinical trials that GLP1-R agonist treatment reduced body weight [7].

GLP-1R agonists first received approval by the US Food and Drug Administration in 2005 (exenatide) and for treatment of obesity in 2015 (liraglutide). More recently semaglutide was approved for treatment of obesity and overweight; compared to the previous GLP-1R agonists, it offers the significant benefits of once-weekly (instead of daily) treatment and a superior capacity to lower body weight (for a detailed review on the history of GLP-1R agonist drugs developments and effects on body weight see [19]). Clinical results indicate that patients with obesity but not T2D receiving semaglutide show body weight reductions in the range of 15% [20]. Recent data from the SELECT trial indicate that in patients living with obesity treated with semaglutide for up to 4 years, an average body weight reduction of 10% is observed [21]. Although these decreases in body weight are smaller than those achieved by surgical procedures, they are highly significant for the clinical management of obesity.

The weight reducing effects of GLP-1R agonists are primarily attributed to their ability to reduce food intake through central mechanisms [22]. GLP-1R is expressed in neurons within the hindbrain and hypothalamus, where agonist-mediated GLP-1R activation translates into decreased food consumption [23]. The consequent reduction in adipose tissue mass largely accounts for the body weight loss induced by GLP-1R agonists. Cohort studies found that the weight loss of liraglutide-treated patients living with obesity is primarily due to a decrease in fat mass, with minimal change seen in lean mass [24]. In the case of semaglutide, a 68-week treatment in individuals living with overweight or obesity yielded an almost 20% reduction in total fat mass [25].

### Effects of GLP-1R Agonists on Adipose Tissue Distribution

Despite the assumption that the reduction in adipose tissue accounts for most of the body weight reduction elicited by GLP-1R agonists, a few studies have measured the sizes of various adipose tissue depots in specific areas following GLP-1R agonist treatments. A recent meta-analysis found that treatment with liraglutide or exenatide reduces the mass of visceral adipose and, to a lesser degree, subcutaneous fat [26]. A recent study reported that liraglutide treatment reduces visceral fat and causes a relative increase in subcutaneous fat deposition in the lower body, potentially reflecting depot-specific alterations in lipid metabolism [27]. Similarly, preclinical studies in rats showed that liraglutide treatment preferentially reduced visceral fat compared to subcutaneous fat [28]. These observations are relevant given that enlarged visceral adipose tissue is primarily responsible for obesity-related complications, while subcutaneous fat is generally considered to be neutral or even protective [29].

A few studies have explored how liraglutide affects adipose tissue properties beyond changes in size. A recent study reported that liraglutide treatment increases the metabolic activity of visceral adipose tissue [30] but another study performed using subcutaneous fat biopsies found that liraglutide did not improve in obesity-related dysfunction, particularly in terms of inflammation and fibrosis within subcutaneous adipose tissue [31]. Meta-analyses, mainly derived from studies on patients with type 2 diabetes, found that liraglutide or exenatide treatment increased adiponectin levels [32] and reduced leptin levels [33], indicating that GLP-1R agonism has concordant effects on the size and secretome of adipose tissue.

Data on the effects of semaglutide on adipose tissue are more limited. The DEXA-based analysis included in the Semaglutide Treatment Effect in People with Obesity (STEP) clinical trial program [34] indicated that semaglutide treatment notably reduces both total fat mass and regional visceral fat mass relative to baseline [20], but this study did not explore potential differential effects on distinct adipose depots.

In addition to the common assessment of the visceral and subcutaneous depots, several studies found reductions in the epicardial adipose tissue of patients receiving liraglutide [35–37] or semaglutide [38, 39]. Epicardial adipose tissue is a type of fat that is closely attached to the myocardium; it plays a key role in cardiometabolic health, exerting harmful effects when it becomes enlarged [40]. A recent study identified a particularly significant reduction in perirenal adipose tissue among individuals with obesity treated with liraglutide, which could conceivably improve obesity-related hypertension [41].

In summary, the ability of GLP-1R agonists to reduce adipose tissue mass, particularly visceral fat, may have a clear impact on promoting metabolic health. A decrease in visceral adipose tissue size can potentially reduce inflammatory signalling and support a healthier adipokine secretion profile, as seen in patients who lose adipose tissue through non-pharmacological methods like lifestyle changes or bariatric surgery [42].

### Mechanisms of Action of GLP-1R Agonism Treatment on WAT

It remains unclear whether GLP-1R agonists have direct effects on adipose tissue, beyond their indirect effects mediated by central mechanisms and reduced food intake. It is generally believed that adipocytes express only minimal levels of GLP-1R; rather, its detection in bulk WAT samples is often attributed to its expression in non-adipocyte cells within the tissue. However, some studies have suggested that GLP-1R can show significant expression in human visceral adipose tissue, particularly in the stromal vascular fraction, which includes mesenchymal stem cells, preadipocytes, fibroblasts, vascular endothelial cells, and various immune cells [43]. While one study reported that white adipocytes express GLP-1R at just over 10% of the level found in tissues that are highly responsive to GLP-1, such as the stomach [43], other research found little to no GLP-1R expression in adipocytes and substantial expression only in non-adipocyte cells within the tissue, such as monocytes [44]. It remains technically challenging to reliably measure GLP-1R levels in cells and tissues; as discussed by Drucker (2018) [45], notably due to the lack of validity of the antibodies available. This may explain the remaining uncertainty regarding the presence of GLP-1R in adipose tissues. Further studies using knockout models in rodents may provide insight into the in vivo effects of deficiencies in these receptors.

Several in vitro studies have shown effects of GLP-1R agonism on human white adipocytes and rodent models of adipogenesis. The results showed that GLP-1R agonists promote the differentiation of preadipocytes into white adipocytes and induce various metabolic effects, including increases in insulin-dependent glucose uptake, lipogenesis, lipolysis, and adiponectin secretion, and a reduction in pro-inflammatory signalling [46–48]. However, the physiological significance of these findings is uncertain, and it remains unclear whether any of the systemic effects elicited by GLP-1R agonists involve a direct action on adipocytes.

**Fig. 1:**
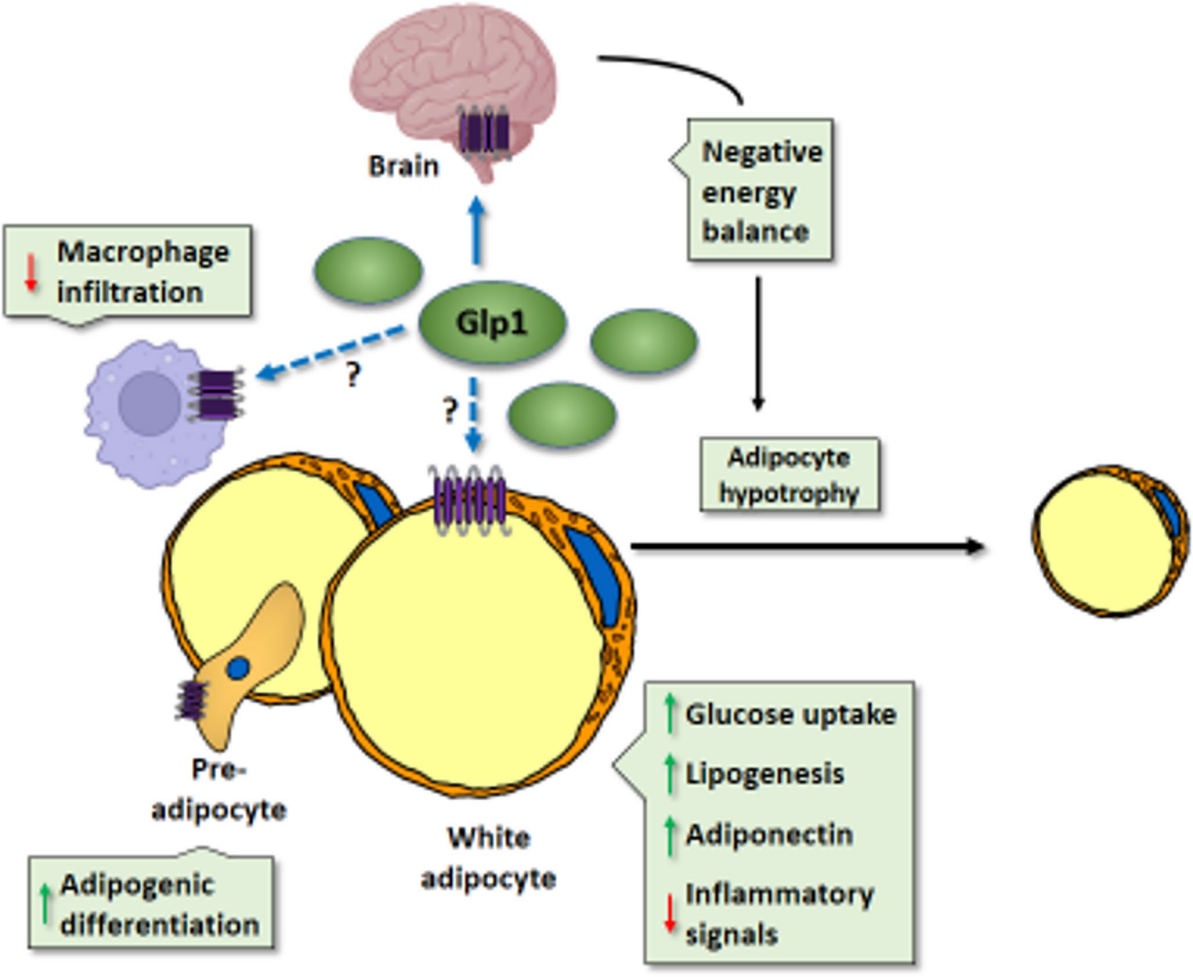
Mechanisms of action of GLP-1 on WAT. GLP-1 targets the central nervous system via the GLP-1 receptor, promoting satiety and resulting in a negative energy balance that reduces adipocyte size and lipid content. Although this is not a consensually accepted view (as indicated by the dotted arrows and question marks), there is evidence proposing that GLP-1 can directly act on macrophages within WAT to reduce inflammation, on pre-adipocytes to promote adipogenic differentiation, and on white adipocytes to regulate metabolism

Local inflammation at hypertrophied WAT is a hallmark of insulin resistance and other comorbidities associated with obesity. GLP-1R agonists reportedly reduce macrophage infiltration and inflammation at WAT in experimental models of obesity [49]. Moreover, recent data indicate that GLP-1R agonist treatment restores the functionally defective natural killer (NKT) cells in people living with obesity, independently of its weight loss effect [50]. Some evidence suggests that immune cells express GLP-1R, raising the possibility that this anti-inflammatory effect could result from a direct action of GLP-1 on non-adipocyte infiltrating immune cells within adipose tissue. However, a recent study indicated that the reduction in systemic inflammation caused by GLP-1R agonism occurs primarily via the central effects of GLP-1 [51]. This recent study did not specifically address obesity-related adipose tissue inflammation, and the potential for GLP-1R agonism to directly influence immune cells within adipose tissue and thereby mitigate local inflammation remains an open question (Fig. 1).

## GIPR Agonism and WAT

### Incorporating GIPR Agonism with GLP-1R Agonism: Effects on Body Weight and Adiposity

Recent years have seen remarkable advancements in obesity treatment achieved by the development of new drugs that combine GLP-1R and GIPR co-agonism in a single molecule. The efforts to develop these dual agonists were initially aimed at improving glucose metabolism in T2D through additive insulinotropic effects and promote greater weight loss by further reducing food intake. However, there is an ongoing debate on whether increasing or decreasing GIPR agonism can benefit body weight and metabolism, in part based on the observation that GIPR-null mice are protected against obesity [52]. The use of GLP-1R agonism plus GIPR antagonism has paradoxically shown positive results similar to those obtained with GLP1-GIPR agonists in preclinical studies. Similar evidence in humans remain in phase I settings [53, 54]. To date, the only FDA-approved drug involving GIPR action, tirzepatide, combines GLP-1R and GIPR agonism. It was approved in 2022 for the treatment of T2D and later in 2023 for the treatment of obesity.

Multiple clinical trials showed that tirzepatide enhances weight loss efficacy and yields impressive reductions in body weight relative to results obtained using a single GLP-1R agonist [55, 56]. In fact, preclinical studies had already shown that molecules combining GLP-1R and GIPR agonism are more effective than GLP-1R agonists alone in promoting weight loss and reducing food intake [57, 58]. A key distinction between agonism of GLP-1R/GIPR agonism and that of GLP-1R alone may lie in the peripheral effects of GIPR agonism, which arise from the broader expression (including in adipose tissue) of GIPR compared to GLP-1R. Mice lacking GIPR in the central nervous system showed complete abolition of the GIP-driven inhibition of food intake but only partial reduction of GIP-induced weight loss [59]. This suggested that GIPR agonism promotes weight loss through both central inhibition of food intake and peripheral mechanisms unrelated to food intake. However, other studies did not find significant effects of GIP on body weight in mice with deletion of GIPR in inhibitory GABAergic neurons [60]. For tirzepatide treatment, only around 20% of the improvement achieved in insulin sensitivity can be attributed to weight loss [61], whereas for single GLP-1R agonists the enhancement in insulin sensitivity is entirely attributable to weight loss [62]. Moreover, experimental tirzepatide treatment exerts systemic anti-inflammatory effects even in mice lacking GLP1R in the brain [51]. These findings have heightened interest in clarifying the role of GIP action on peripheral tissues, particularly adipose tissue, and whether such effects mediate some of the pharmacological effects of GIPR agonism.

### Effects of GIPR Agonism in WAT

Several clinical trials showed that tirzepatide treatment significantly reduces the masses of total fat and visceral adipose tissue. Compared to treatments based on single GLP-1 receptor agonism (e.g., semaglutide) over the same duration, tirzepatide demonstrated superior decreases in both total and visceral adipose tissue [63]. In patients living with T2D, tirzepatide treatment was reported to preferentially reduce visceral over subcutaneous adipose tissue, suggesting that the treatment triggered a shift toward a healthier fat distribution pattern [64]. However, another study reported similar decreases in visceral and subcutaneous abdominal fat following tirzepatide treatment [65].

### Cellular Basis of GIP Action on WAT

In rodents and humans, WAT expresses the transcript encoding GIPR [66]; the level is higher in visceral adipose tissue compared to subcutaneous adipose tissue, and the GIPR transcript is downregulated under insulin resistance [67]. There is a debate regarding which cells within WAT are responsible for this expression. Some studies suggest that GIPR is predominantly expressed in non-adipocyte cell types within WAT [68], while others indicate that white adipocytes from both mice and humans express significant levels of GIPR, even though still lower than in the stromal vascular fraction [69], but this expression is induced during adipocyte differentiation [70]. A recent study has convincingly demonstrated that a fluorescently labeled selective GIPR mono-agonist (GIPRA-FL) associates with adipocyte membranes, and GIPR expression in adipocytes was shown to mediate intracellular signaling in response to GIP, thereby regulating functional cellular responses [69]. These findings support a direct role for GIP in modulating adipocyte function. The study further reveals that the dual GIPR/GLP-1R agonist tirzepatide directly associates with adipocytes and activates intracellular signaling specifically through GIPR engagement. This functional GIPR expression, responsive to both tirzepatide and GIP, has been demonstrated in both human and mouse adipocytes. Collectively, these findings indicate the presence of active GIPR-dependent signaling in white adipocytes and suggest that tirzepatide may modulate adipocyte function through GIPR agonism. Moreover, multiple studies show that GIP treatment of adipocytes in vitro exerts direct metabolic effects, including increased insulin sensitivity and glucose uptake [71, 72]. Recent data based on single nuclei RNAseq analysis of WAT confirmed substantial expression of GIPR transcript in adipocytes [73].

GIPR agonism affects glucose metabolism in WAT. In the absence of GLP-1R, treatment with tirzepatide or the long-acting GIPR agonist, LAGIPRA, improves insulin sensitivity by enhancing glucose disposal in WAT; moreover, at least some degree of the tirzepatide-induced improvement of insulin sensitivity occurs to some extent in a weight-independent manner [74]. This aligns with the evidence showing that GIP can increase glucose uptake in adipose tissue [69].

GIP significantly affects lipid metabolism in adipocytes, promoting lipolysis [75] and enhancing lipoprotein lipase activity [76, 77]. It also promotes fatty acid re-esterification [78, 79]. Tirzepatide shows similar effects, increasing the hydrolysis of dietary triglycerides and fatty acid uptake in adipose tissue [69]. Although the role of GIP in fat metabolism may seem contradictory to its ability to reduce fat mass, recent views suggest GIPR agonism supports healthy WAT expansion by clearing triglycerides and preventing lipotoxicity, alongside its ability to reduce fat mass through lower food intake, and thereby reduces insulin resistance and other obesity-related comorbidities [80].

**Fig. 2:**
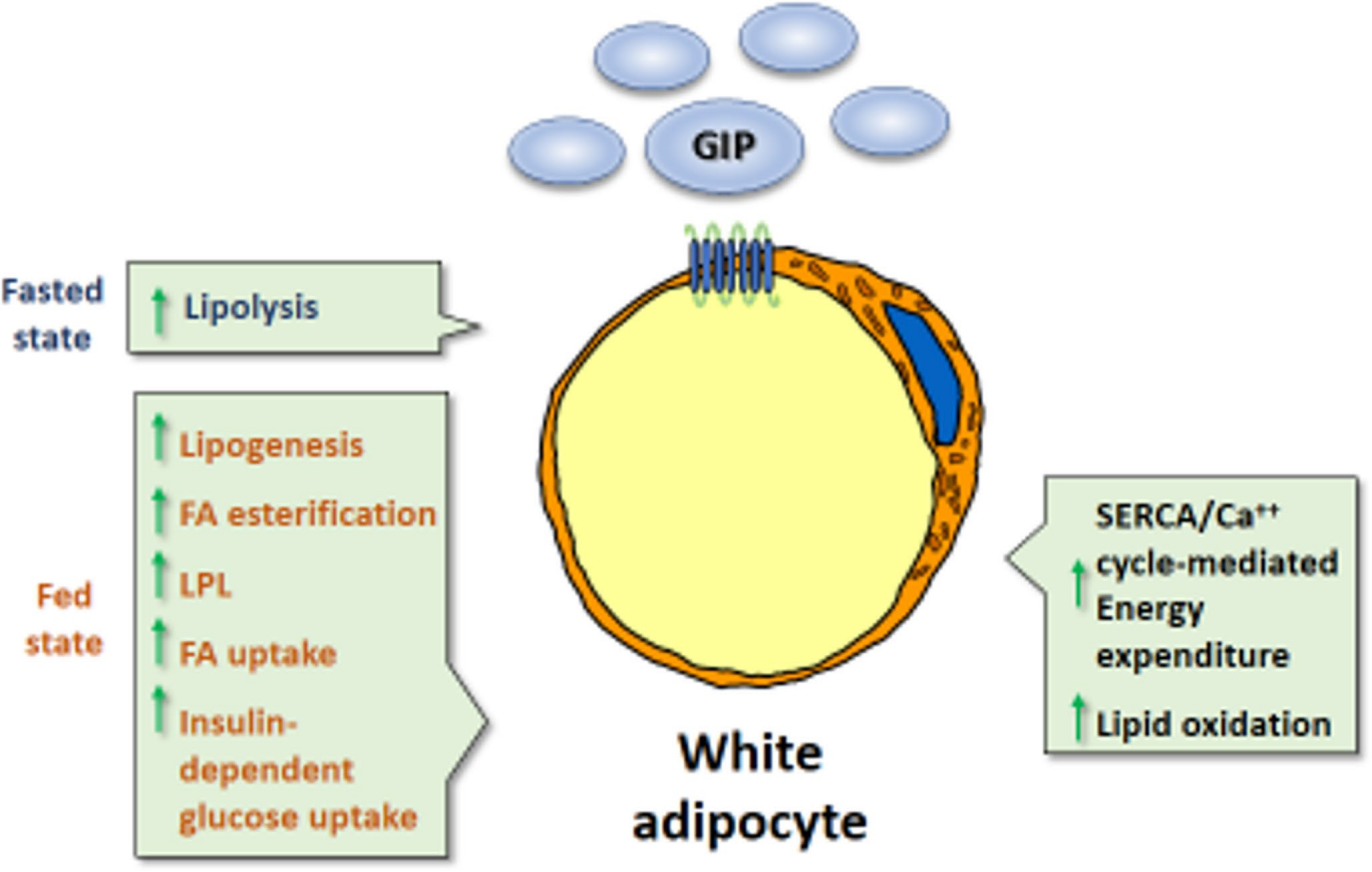
Mechanisms of action of GIP on WAT. GIP can target pre-adipocytes and adipocytes via GIPR. In adipocytes, GIP promotes insulin-dependent glucose uptake and alters lipid metabolism distinctly according to the fed (high insulin levels) or fasted (low insulin levels) state. It also promotes lipid oxidation in the white adipocyte to increase whole body energy expenditure via activation of the SERCA-driven futile calcium cycle. FA, fatty acid, LPL, lipoprotein lipase

A recent study proposed that GIPR agonism can have differential effects on adipose tissue and metabolism depending on the feeding status, and this is linked to the action of insulin [69]. In the fed state, which is characterized by high insulin levels, GIPR agonism promotes triglyceride clearance by increasing lipoprotein lipase activity, along with fatty acid and glucose uptake. This contrasts with the fasting state, wherein insulin levels are low and GIP action inducing lipolysis predominates (Fig. 2). It should be noted that tirzepatide-induced GIP differs from natural GIP stimuli, in that the former has a prolonged action that extends beyond the postprandial period into the post-absorptive phase, whereas the effects of natural GIP occur only in the post-meal period. This sustained pharmacological GIPR agonism has been proposed to govern the non-obesogenic and even leaning effects embodied by the GIPR agonism in the dual GLP-1R-GIPR agonists. GIP acts on its cellular targets by binding to GIPR, triggering intracellular Gαs-mediated cAMP production and β-arrestin recruitment [81]. Recent studies identified human GIPR variants with differing abilities to activate these two signalling pathways and showed that the balance between them appears to influence GIPR agonism effects. For example, a variant associated with lower cAMP and β-arrestin signalling is strongly linked to reduced adiposity. It has been suggested the GIPR agonism of tirzepatide may mimic the actions of native GIP but with reduced recruitment of β-arrestin recruitment to GLP-1R but full activation of the cAMP pathway, thereby contributing to the ability of tirzepatide to decrease adiposity [82, 83].

Finally, a recent study highlights the direct effects of GIPR agonism in promoting energy expenditure in WAT [73]. Several preclinical and clinical data suggest a role for GIPR agonism, as seen in dual GLP-1/GIPR agonists, in enhancing energy expenditure as part of their anti-obesity effects. This has led to investigations into their potential role in BAT activation and the browning of WAT (see Sect. 5). Yu and collaborators reported that GIPR agonism protects against obesity in mice by inducing thermogenesis in WAT itself through the promotion of the Sarcoendoplasmic Reticulum Calcium ATPase (SERCA)-driven futile calcium cycle [73]. Specifically, the authors used an inducible adipose tissue-specific mouse model of GIPR overexpression, which enabled them to identify a mechanism whereby GIPR induction exclusively in the white adipocyte promotes activation of SERCA-mediated futile calcium cycling, to increase adipose tissue lipid oxidation and whole body energy expenditure. This metabolic pathway is based on an ATP-hydrolyzing mechanism elicited by futile calcium cycling, originally known to favor energy expenditure in muscle and beige adipocytes [73, 84]. Very recently, Kajimura and colleagues demonstrated that microsomal thermogenesis in adipocytes modulates SERCA2b-mediated Ca²⁺ import [85], a process that plays a central role in UCP1-independent thermogenesis and energy homeostasis. This finding further underscores the relevance of futile calcium cycling in adipocytes, particularly in the context of its modulation by GIPR agonism, as discussed above.

## Glucagon Receptor Agonism and Obesity Treatment

### Incorporating Glucagon Receptor Agonism To Drugs for Obesity Treatment: Implications for Adiposity

Glucagon receptor (GCGR) recently emerged as an additional target in the development of anti-obesity drugs. Beyond its role in pancreatic function, GCG has gained recognition as a regulator of energy balance, and several studies have explored its potential as a weight-regulatory hormone. In preclinical animal models, GCGR agonism has been shown to reduce food intake and especially to increase energy expenditure, contributing to weight loss [86]. GLP-1R/GCGR unimolecular co-agonists have been developed to leverage the complementary pharmacological benefits of GCG’s powerful promotion of energy expenditure and GLP-1’s anorectic (appetite-reducing) and glucose-lowering effects, while using the presence of GLP-1R agonism to minimize potential adverse effects of GCGR agonism (such as glycaemic effects leading to diabetogenic risks). On preclinical studies, several GLP-1R/GCGR co-agonists have demonstrated superior weight loss, glucose regulation, and food intake reduction effects compared to those of single GLP-1R agonists [87–89]. Some of these co-agonists have moved into clinical development, where they yield notable improvements in body weight and often improved comorbidities such as hypertension [90] or metabolic dysfunction-associated steatohepatitis [91]. Although no GLP-1R/GCGR co-agonist is yet approved for clinical use, a number are currently under phase 2 or phase 3 trials for treatment of obesity and comorbidities (for updated review see [56]).

Researchers next sought to combine agonists for GCGR, GLP-1R, and GIPR into a single molecule (a tri-agonist) that activates all three receptors [92], each providing unique metabolic benefits: GLP-1R reduces food intake and improves glycaemic control, GCGR boosts energy expenditure, and GIPR may enhance GLP-1’s satiety effects and improve insulin sensitivity. Several tri-agonist peptides have been developed to date [93]; they show superior results in regulating body weight, liver lipid metabolism, and glycaemic control compared to existing treatments. Early clinical trials of some tri-agonists (e.g. retatrutide) have yielded significant body weight reductions (24–28%) in individuals living with obesity after 48 weeks of treatment [94]. Clinical trials of dual or triple agonists involving GCGR agonism, have not specifically provided data on the impact of these drugs on adipose distribution and properties but fat loss is generally believed to drive the body weight reductions triggered by these drugs.

### Glucagon and WAT

The role of GCG in WAT has not been extensively studied; to date, the research has focused more on its energy expenditure-increasing effects potentially mediated by BAT (see below). WAT expresses GCGR, but at lower levels than seen in the liver [95, 96]. GCG was thought to induce lipolysis in WAT via G-protein coupled receptor action, but recent studies in mice with adipose-specific GCGR invalidation failed to show any significant metabolic effect [97]. GCG has been reported to induce lipolysis in vitro in isolated adipocytes but there is some question as to the physiological relevance of these studies, which often used high doses of GCG [98–101]. Moreover, a microdialysis study of subcutaneous adipose tissue in humans found that GCG had no significant effect on lipolysis [102]. In summary, it remains unclear whether GCG action has direct effects on WAT, with recent data supporting mainly indirect effects. In this sense, experimental evidence indicates that GCG targets the liver where it promotes the secretion of fibroblast growth factor-21(FGF21) [103, 104], a hormonal factor involved in regulating energy balance by targeting both the central nervous system and peripheral tissues, including WAT and BAT (see below). Therefore, it cannot be ruled out that GCG exerts indirect effects on WAT by promoting the secretion of FGF21, and possibly other regulatory hepatokines, that can target WAT.

## A Role for Thermogenic Plasticity of Adipose Tissue in the Actions of incretin-based anti-obesity Drugs

### Thermogenic Plasticity of Adipose Tissue in Obesity and Metabolic Health

BAT is a type of adipose tissue specialized in heat production and is associated with energy expenditure, in contrast to the metabolic energy-storing function of WAT. The thermogenic activity of BAT is driven by uncoupled mitochondrial oxidation in brown adipocytes via the UCP1 protein, stimulated by the sympathetic nervous system and endocrine factors in response to temperature or diet [105, 106]. Rodent studies show that active BAT helps prevent obesity by increasing energy expenditure and aiding in the removal of glucose and lipids from the bloodstream [107–109]. BAT also secretes brown adipokines that affect various tissues systemically [110]. Active BAT’s role in adult humans was discovered around 15 years ago, and though its role in obesity protection is debated, studies have linked it to reduced obesity, type 2 diabetes, and cardiovascular disease [111]. WAT can also develop a thermogenic phenotype through “browning,” [112], a process by which “beige” adipocytes, showing a brown adipocyte-like multilocular morphology and thermogenic capacity mediated by UCP1 and futile cycles, appear in WAT depots [113–115]. Given that the thermogenic plasticity of adipose tissue is important for energy expenditure and metabolic homeostasis, BAT activation and WAT browning have been widely targeted in the development of anti-obesity drugs. However, the reliance on sympathomimetics for this purpose has resulted in their systematic rejection to date due to harmful cardiovascular side effects [116]. With the emergence of incretin agonism-based drugs researchers have begun exploring how much of their effects are embodied through promoting activities of thermogenic adipose tissues.

### Effects of GLP-1R Agonism on the Thermogenic Plasticity of Adipose Tissue

#### GLP-1R Agonists Promote BAT Activity and WAT Browning in Preclinical Models

Several preclinical studies in mouse models have suggested that the weight loss induced by GLP-1R agonists is not due solely to their anorectic effects but also may involve changes in energy expenditure that occur through the activation of BAT and/or the recruitment of beige adipocytes in WAT [117]. This happens largely via indirect mechanisms that are mediated by central GLP-1R activation and at least partially depend on sympathetic nervous system signalling to thermogenic adipose tissues [118, 119]. Thus, administration of GLP-1R agonists into the hypothalamic ventromedial nucleus increases sympathetic nervous system activity leading to enhancement of BAT thermogenesis and browning of WAT [120]. Intracerebroventricular injection of exendin-4 similarly increased sympathetic outflow to both BAT and WAT, resulting in higher thermogenesis in mice [121]. Intraperitoneal administration of liraglutide also reportedly increased oxygen consumption, UCP1 levels and type 2 deiodinase activity in BAT and subcutaneous WAT [122]. These results collectively underscore BAT activation and WAT browning driven by central effects of GLP-1R agonism and subsequent sympathetic activation; however, further studies are needed to establish the role of these effects in the systemic metabolic and body weight changes in response to GLP-1R agonism-based drugs.

#### Human Studies Addressing the Effects of GLP-1R Agonism on BAT

Unlike the findings from preclinical studies, the evidence available from humans suggests that GLP-1R agonist-induced weight loss is primarily mediated by reduced appetite and energy intake rather than increased energy expenditure [123]. A systematic review and meta-analysis [124] concluded that the GLP-1R agonists, exenatide and liraglutide, have neutral effects on resting energy expenditure.

To date, only two studies have investigated the potential effects of GLP-1R agonists on human BAT [125, 126]. The first such study found that 26-week liraglutide treatment of patients living with T2D did not reduce the fat fraction in BAT (a surrogate of BAT activity) or alter resting energy expenditure [125]. The second study found that 12 weeks exenatide treatment reduced body weight without affecting resting energy expenditure but increased the metabolic volume and glucose uptake in both cervical and supraclavicular BAT [126], as measured by [18 F] fluorodeoxyglucose positron emission tomography, which is the gold-standard method for measuring BAT activity in humans [127]. This significant finding in young, non-diabetic, lean adults, supports the idea that GLP-1R agonism contributes to activating BAT. Moreover, a recent case report highlighted increased BAT activity in a woman with obesity treated with the GLP-1R agonist semaglutide [128]. Further studies specifically evaluating BAT activity in individuals living with obesity treated with GLP-1R agonist-based therapies are needed to draw firm conclusions on BAT activation in patients.

#### Immune cell-based Mechanisms Contributing To the Effects of GLP-1R Agonism on Thermogenic Adipose Tissue

As discussed earlier, it remains unclear whether GLP-1R agonists act directly on adipose tissues, including thermogenic fat. However, recent preclinical studies have shed new light on the involvement of immune cells in the thermogenic activation of adipose tissues observed under GLP-1R agonism [44, 129]. The results from these studies suggest that GLP-1 signalling may target immune cells, such as monocytes/macrophages within adipose tissues, which could directly affect adipose tissue functions. In mice, peripheral administration of liraglutide activates invariant NKT (iNKT) cells in WAT, increasing FGF21 production to promote the browning of inguinal WAT and contribute to weight loss [129]. Indeed, it has been shown that FGF21 is required for weight loss induced by liraglutide in male mice [130]. In iNKT-deficient mice, liraglutide promotes satiety but fails to induce FGF21, leading to reduced weight loss. Remarkably, humans treated with liraglutide also exhibit increased peripheral levels of iNKT cells and FGF21, both of which correlate with the degree of weight loss induced by the therapy [129]. A recent study revealed that intraperitoneal liraglutide administration in mice leads to a transient upregulation of monocyte interleukin-6 (IL-6) secretion, raising circulating IL-6 levels and directly targeting IL-6 receptor signalling in preadipocytes/adipocytes. The ability of liraglutide to induce energy expenditure, brown adipogenesis and thermogenesis requires the presence of active IL-6 receptor [44]. Interestingly, exenatide treatment in humans living with prediabetes also acutely induced IL-6 secretion by monocytes and increased systemic IL-6 levels [44]. Although IL-6 can be considered a pro-inflammatory cytokine, it may have positive functions for metabolic health depending on its site of synthesis and secretion [131]. In fact, IL-6 acts as a “batokine,” when its secretion by brown adipocytes is induced in response to sympathetic stimulation [132].

**Fig. 3:**
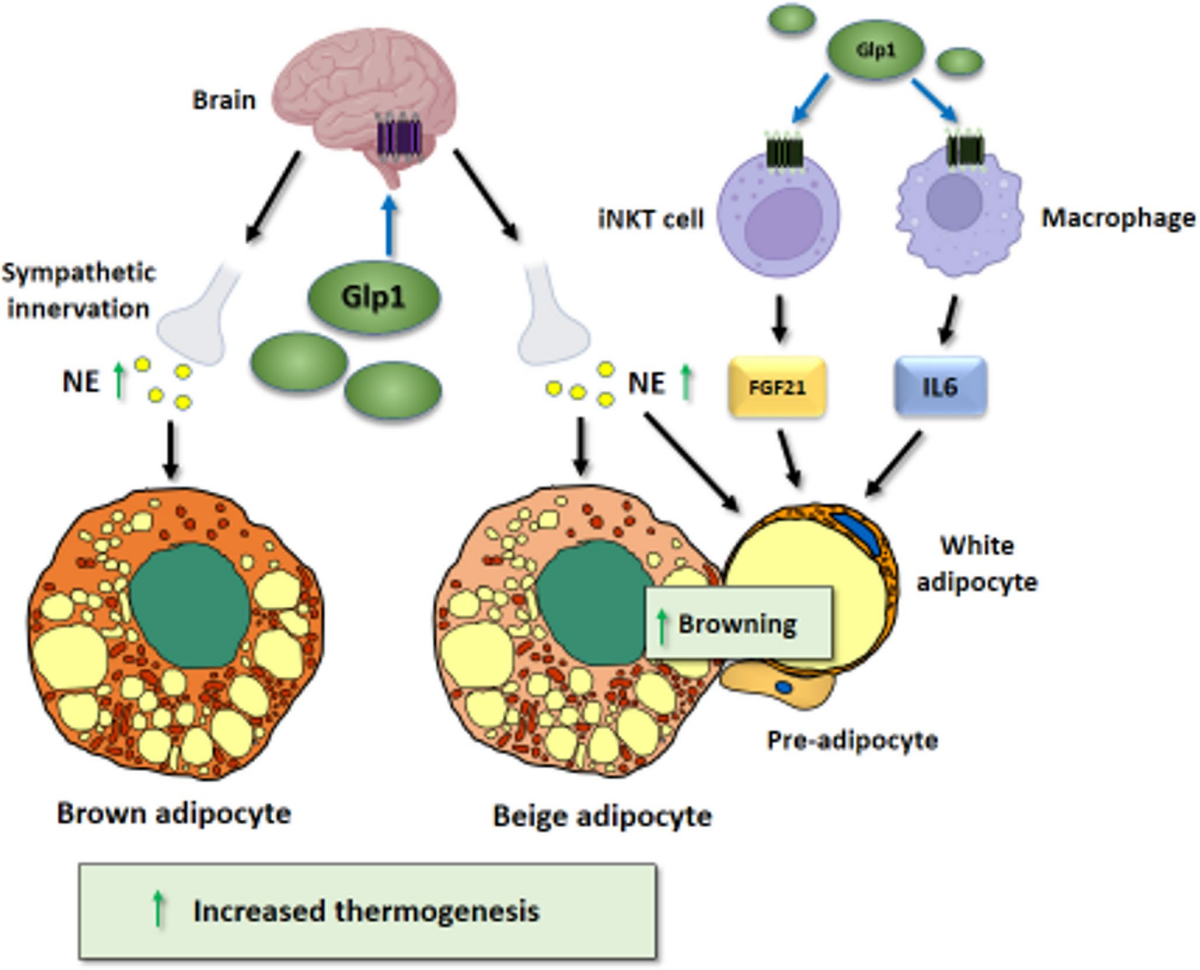
Mechanisms of action of GLP-1 on BAT and WAT browning. GLP-1 targets the central nervous system through the GLP-1 receptor, which activates the sympathetic nervous system and induces noradrenergic signalling to BAT and browning-prone WAT. Additionally, GLP-1 interacts with GLP-1R on invariant natural killer T (iNKT) cells and macrophages, stimulating the secretion of FGF21 and IL-6, both of which promote WAT browning. NE, norepinephrine

The results of these studies emphasize that crosstalk between immune cells and adipocyte lineages capable of adopting beige and/or brown thermogenic phenotypes is important for the ability of GLP-1R agonism to promote the thermogenic plasticity of adipose tissue (Fig. 3).

### Effects of GIPR Agonism on Brown Adipose Tissue and Adipose Tissue Browning

#### Multi-receptor Drugs Containing GIPR Agonism Activate BAT and Promote Adipose Tissue Browning in Preclinical Studies

Following the incorporation of GIPR agonism activity into dual receptor agonist drugs (e.g. tirzepatide), several studies have investigated whether such activity impacts BAT thermogenic activity and WAT browning to promote energy expenditure and thereby contribute to weight loss. Preclinical studies in mice showed that the positive effects of dual GLP-1R/GIPR agonism on body weight reduction in mice include a significant increase in energy expenditure [58]. Among humans, patients treated with semaglutide or tirzepatide and having comparable energy intake, the latter lose more weight, potentially through enhancement of energy expenditure [133]. Although the literature currently lacks direct robust data on how tirzepatide impacts energy expenditure in patients, preliminary data from the NCT04081337 trial have been used to suggest that tirzepatide may attenuate the adaptive reduction in energy expenditure occurring in association with weight loss [134].

Experimental studies in diet-induced obese mice have shown that tirzepatide treatment enhances glucose uptake, upregulates genes associated with thermogenic activation and metabolic catabolism [74] and induces a thermogenic-like amino acid signature [135] in BAT. Tirzepatide administration in mice can decrease the circulating levels of branched-chain amino acids (BCAA) and ketoacids [74], and defective BCAA catabolism in thermogenic adipose tissues can impair thermogenesis [136]. Notably, the effects of tirzepatide in individuals living with T2D include significant reductions in BCAA and related metabolites [137]. The effects of tirzepatide on BAT and the browning of WAT have been attributed to the GIPR agonism component, as these effects of tirzepatide are largely mimicked by treatment with the long-acting GIPR agonist LAGIPRA [74, 138]. However, this assumption, based on mouse models, should be approached with caution, given the evidence that, unlike in humans, tirzepatide is less potent at the mouse GIPR than mouse GIP [139]. To date, the effects of GIPR agonism on BAT have not yet been thoroughly assessed in humans. However, while acute GIP infusion does not appear to alter energy expenditure in humans [140], 6 days of subcutaneous GIP infusion can increase the temperature of the supraclavicular area, which is a primary site of BAT depots [141].

#### Mechanisms Through Which GIPR Agonism Promotes Adipose Thermogenic Plasticity

Although studies using tirzepatide and other molecules with GIPR agonism generally show thermogenic activation of BAT and WAT, research involving genetic invalidation of GIPR has yielded somewhat contradictory results. This appears analogous to the paradoxically similar abilities of GIPR agonist treatment and genetic invalidation of GIPR to protect mice against diet-induced obesity.

The GIPR transcript is expressed in BAT at levels similar to those seen in other adipose depots [66]. This expression arises preferentially in the brown adipocyte fraction of the tissue [66], but GIPR is also expressed in other cell types (e.g., immune cells) within BAT and in browning-prone WAT depots. The literature currently lacks solid data on GIPR protein levels in distinct cell types within thermogenic adipose tissue.

Selective loss of GIPR within BAT (but not in potentially thermogenic inguinal WAT) does not significantly alter energy expenditure or metabolism in mice under feeding of an obesogenic diet [66]. In contrast, invalidation of GIPR in myeloid cells decreases energy expenditure, inhibits the WAT browning [142], increases inflammation, and reduces the synthesis of type 2 cytokines within adipose tissue [143]. This aligns with the known role of inflammation in repressing adipose thermogenic activity and the importance of type 2 immunity in activating BAT and beige adipose tissue [144]. In vitro studies have shown that GIP treatment increases the expression of the thermogenic marker gene *UCP1* and the secretion of IL-6. However, paradoxically, GIPR gene knockout also leads to increased expression of *UCP1* and other genes related to BAT thermogenesis and metabolism [66]. Another study found that GIP treatment increases the browning of in vitro cultured adipose precursor cells from omental WAT[145].

**Fig. 4:**
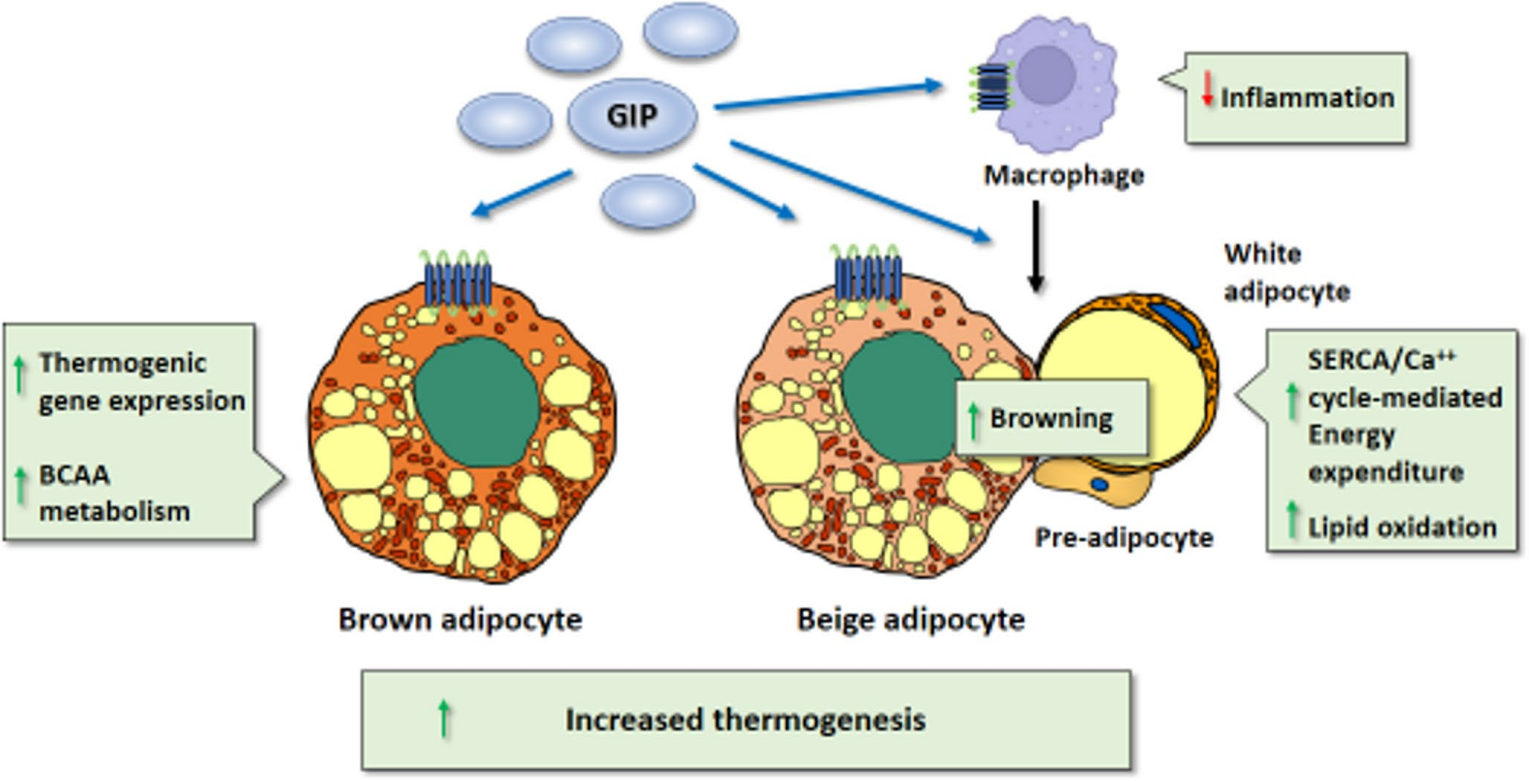
Mechanisms of action of GIP on BAT and WAT browning. GIP targets GIPR in macrophages, resulting in reduced inflammation and increased synthesis of type 2 cytokines, which promote WAT browning. Additionally, GIP induces the thermogenic activation of adipose tissue by interacting with GIPR in brown and beige adipocytes, while also promoting the browning of WAT by acting on pre-adipocytes. Additionally, white adipocytes contribute to energy expenditure via lipid oxidation and activation of the SERCA-driven futile calcium cycle (see 3.3)

In summary, it appears that GIPR agonism has a widespread capacity to induce the thermogenic plasticity of adipose tissue by targeting distinct cell types within the tissue, such as brown adipocytes, beige adipocytes and especially immune cells, which are key mediators of thermogenic remodelling of adipose tissue (Fig. 4). As mentioned above, a recent report demonstrated that GIPR can induce lipid oxidation and thermogenesis in white adipocytes to increase energy expenditure by promoting SERCA-calcium futile cycling, even in the absence of a clear acquisition of beige morphology [73].

### The Potential of GCGR Agonism in Promoting Adipose Tissue Thermogenic Plasticity

#### Glucagon Agonism as a Potential Tool To Promote Energy Expenditure in incretin-based Treatments against Obesity

The rationale for incorporating GCGR agonism into multi-receptor incretin-based drugs stems from findings indicating that GCG can increase energy expenditure. For example, GCG has been shown to induce energy expenditure in various preclinical animal models (see [86] and [146] for review), offering protection against diet-induced obesity. In humans, GCG infusion has been reported to increase energy expenditure in both lean people and individuals living with obesity [147, 148], although not all studies have confirmed this effect [149]. Limited data have directly confirmed that GCGR agonism in dual (GLP-1R/GCGR) or triple (GLP-1/GIP/GCGR) agonist drugs provide the same benefits promoting energy expenditure. In reports on experimental studies, the weight loss achieved by the GLP-1R/GCG co-agonist MEDI0382 has been partially attributed to GCGR-dependent induction of energy expenditure [150]. In humans, GCG has shown a strong capacity to induce energy expenditure when combined with a single GLP-1R agonist in individuals living with obesity [151]. Recent data on individuals living with overweight and obesity treated with the GLP-1R/GCGR co-agonist SAR425899 indicated that such individuals exhibit a smaller-than-expected decrease in the sleeping metabolic rate, increased fat oxidation but no overt induction of energy expenditure [152].

#### Effects of Glucagon Agonism on BAT and WAT Browning and the Relationship with Energy Expenditure

Accumulated evidence indicates that GCG treatment activates BAT thermogenesis in preclinical models, in association with increased energy expenditure (for review see [86]). The relevant effects of GCG include upregulation of UCP1 expression and sustained effects on BAT recruitment, including mitochondrial biogenesis and hypertrophy of thermogenically active BAT. Recently, GCG was reported to induce WAT browning in a GCGR-dependent fashion [153]. Furthermore, a study reported that treating mice with the dual GLP-1/GCG agonist cotadutide activates BAT thermogenesis; however, the relative contributions of GLP-1 and GCG agonism was not assessed [154]. Recently, a superior efficacy of dual GLP-1R/GCGR agonism versus single GLP-1 agonism in reducing obesity in mice has been attributed to a remodelling of WAT towards browning and UCP-1-dependent BAT activation [155].

However, the role of BAT activation in GCG energy expenditure-promoting effects is less clear. Studies in mice with whole-body GCGR invalidation concluded that GCG is essential for adaptive thermogenesis in BAT [156]. However, Myf5 promoter-driven deletion of GCGR in BAT did not affect diet-induced obesity or glucagon-induced energy expenditure [96]. These data suggested that GCGR in BAT is not essential for energy homeostasis in mice; however, thermogenically active beige adipocytes are unlikely to be affected in mice with Myf5 promoter-driven deletion of GCGR as they do not develop from a Myf5-expressing cell lineage [157]. GCG can still increase energy expenditure in Ucp1-null mice [96] maybe because BAT and beige adipose tissue include thermogenic cells that do not express UCP1 [114]. In humans, the only available data indicate that GCG infusion increases energy expenditure but does not significantly increase BAT activity [158].

#### Action Mechanisms of Glucagon on BAT and WAT Browning

Substantial evidence indicates that GCG may exert direct effects on BAT. GCGR is expressed in BAT, though at lower levels than in the liver and WAT [96]. In vitro studies have shown that GCG stimulates lipolysis, lipid oxidation, and oxygen consumption in BAT explants [96]. Cultured brown adipocytes treated with GCG exhibit upregulation of thermogenic genes and lipolysis and these effects are impaired when GCGR is invalidated [96]. Consistent with these direct effects, GCGR activation increases intracellular cAMP, which is a major mediator of thermogenic activation in brown and beige adipocytes. GCG also exerts indirect effects by promoting BAT activation and WAT browning through the activation of hepatic FGF21 expression and release. FGF21 is a powerful inducer of thermogenic activation in adipose tissues [159, 160], and results obtained from FGF21-null mice showed that FGF21 critically mediates the effects of GCG on BAT activity and WAT browning [103, 104]. GCG also induces the hepatic production of bile acids, which are known activators of BAT [161] to further contribute to the thermogenic plasticity of adipose tissue [103]. It has been proposed that action mechanism of GCG may differ by the duration of the stimulus: the acute effects of GCG are only partially dependent on BAT activation whereas the long-term effects are largely embodied by the indirect actions of GCG on BAT via FGF21 and bile acids [16].

**Fig. 5:**
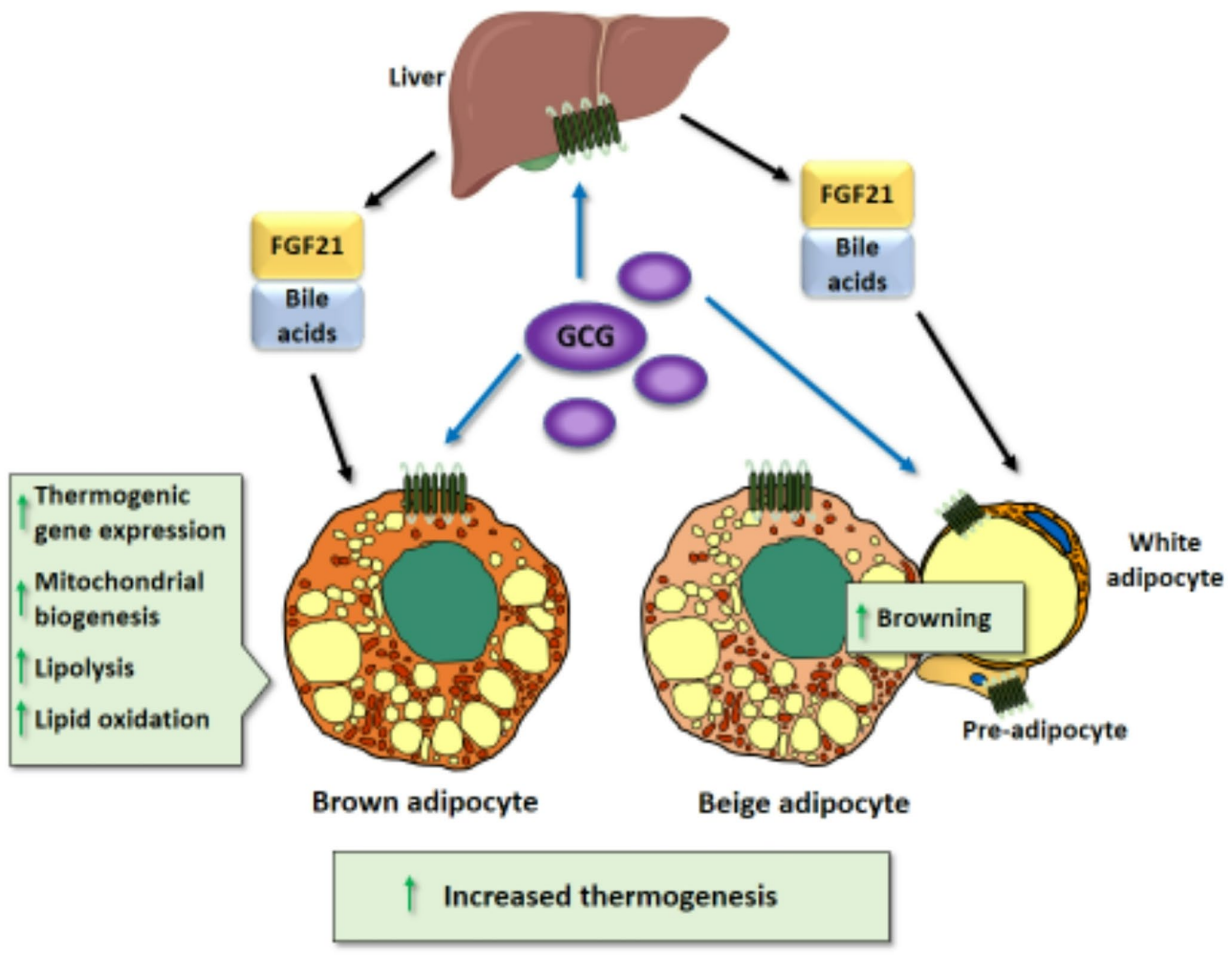
Mechanisms of action of GCG on BAT and WAT browning. GCG acts on GCGR in brown and beige adipocytes inducing thermogenic gene expression and metabolic processes associated with active thermogenesis. In addition, GCG promotes BAT thermogenic activity and WAT browning indirectly through its action in the liver leading to the secretion of FGF21 and bile acids, known inducers of thermogenic activation of adipose tissues

In summary, the experimental data strongly supports idea that GCGR agonism increases BAT and beige thermogenesis through several mechanisms while also increasing energy expenditure, although the cause-and-effect relationship of these two processes is not clear. Further research is needed to ascertain whether the GCGR agonism present in multi-receptors anti-obesity drugs under development retains these effects of single GCGR agonism (Fig. 5).

## Concluding Remarks and Perspectives

In conclusion, a growing body of experimental and clinical data indicates that changes occurring in adipose tissue may play a crucial role in mediating the beneficial effects of anti- obesity drugs based on incretin agonism. These effects seem to extend beyond the mere consequences of the fat mass reduction associated with the negative energy balance resulting from reduced food intake. Rather, the drugs appear to affect adipose tissue function either (1) indirectly, through central mechanisms that involve signalling via the peripheral nervous system to adipose and appear to be the predominant effect in GLP-1R agonism; or (2) through direct actions on adipocytes or non-adipocyte cells, such as pre-adipocytes or immune cells within the tissue. This latter mechanism seems particularly relevant for the GIPR agonism and possibly GCGR agonism actions present in the drugs currently under development. Furthermore, there is evidence that the combined GLP-1R/GIPR agonism enhances the thermogenic plasticity of adipose tissue, leading to beneficial effects on energy balance and systemic metabolism.

For the anti-obesity drugs currently in use and under development, several open questions remain regarding their effects on adipose tissue and their broader health implications, which require further research. One important area of investigation is how various treatments influence fat depots, particularly visceral fat, and whether these treatments can fully reverse its obesity-related pro-inflammatory characteristics. Additionally, the impact of treatments on specific adipose depots, such as perivascular adipose tissue, deserves further exploration, especially in light of emerging evidence regarding the reduction of epicardial fat mass and its cardiovascular implications.

On the other hand, a major goal in enhancing current obesity treatments is to prevent the loss of lean body mass during weight loss [162]. The importance of research on the actions of incretin-based drugs in adipose tissue is not unrelated to this challenging issue, in light of the growing understanding of the cross-talk between adipose and muscle tissue through metabolic and endocrine signals [163]. Enhancing the beneficial effects of incretin-based drugs on the adipose tissue secretome and its signaling to muscle may help preserve muscle mass and function. Further research is needed to advance in this field given the importance of elucidating how treatments influence the adipose tissue secretome, a key mediator of systemic inter-organ communication.

Another crucial area of research is examining how different drugs might affect adipose tissue in a gender-specific manner, given the known sexual dimorphism in adipose tissue distribution. This could have significant implications for the prescribing of treatments.

A further critical area of exploration will be confirming in humans the potential for treatments to induce thermogenic plasticity in adipose tissue, a process suggested by preclinical studies, and understanding how different agonists may contribute to enhancing this process.

Lastly, the ability of incretin agonism-based drugs to restore adipose tissue to a healthy state and the persistence of their effects warrants examination. Maintaining weight loss after obesity is challenging, and evidence suggests that non-pharmacological interventions, such as food restriction and bariatric surgery, do not fully reverse obesity’s effects on adipose tissue, particularly in terms of innate immunity and mitochondrial changes in visceral fat [164, 165]. Recent findings attribute this “obese memory” to stable obesity-driven epigenetic changes in adipocytes [166]. Future research is needed to investigate whether drug-based treatments can more completely restore adipose tissue health following weight loss. Recent preclinical data suggesting that GIPR agonism promotes a “healthy metabolic memory” in adipocytes [73] offers promising insights into the potential long-term efficacy of incretin-based drug treatments.

In summary, beyond recognising the significant effects of new anti-obesity drugs in reducing weight and adipose mass, the field currently needs a new level of research to determine the specific actions and consequences of these drugs on the properties of adipose tissue. Such studies should focus not only on changes in tissue mass but, more importantly, on alterations in the biological properties of adipose tissue in treated patients. This work, which will require extensive preclinical research and studies on human cohorts undergoing treatment, could lead to a better understanding of both the drugs currently in use and those in development, and may inspire relevant improvements based on their effects on adipose tissues.

## Key references

- Holst JJ. GLP-1 physiology in obesity and development of incretin-based drugs for chronic weight management. *Nat Metab*. 2024;6:1866–85

- This is an updated review of the current understanding of GLP-1 effects and its role in the use of GLP-1 agonists as anti-obesity drugs.

- Müller TD, Adriaenssens A, Ahrén B, Blüher M, Birkenfeld AL, Campbell JE et al. Glucose-dependent insulinotropic polypeptide (GIP). *Mol Metab*. 2025, 95:102118

- Updated comprehensive review on the multifaceted nature of GIP biology and its therapeutic implications

- Wong CK, McLean BA, Baggio LL, Koehler JA, Hammoud R, Rittig N, et al. Central glucagon-like peptide 1 receptor activation inhibits Toll-like receptor agonist-induced inflammation. *Cell Metab.* 2024;36:130-143.

- The central effects of GLP-1 lead to the downregulation of systemic inflammation.

- Kusminski CM, Perez-Tilve D, Müller TD, DiMarchi RD, Tschöp MH, Scherer PE. Transforming obesity: The advancement of multi-receptor drugs. *Cell*. 2024; 187:3829-3853.

- This is a comprehensive review of the current knowledge on the effects of multi-receptor anti-obesity drugs and their known mechanisms of action.

- Regmi A, Aihara E, Christe ME, Varga G, Beyer TP, Ruan X, et al. Tirzepatide modulates the regulation of adipocyte nutrient metabolism through long-acting activation of the GIP receptor. *Cell Metab*. 2024;36:1534-1549.

- Tirzepatide, due to its GIP agonist component, directly affects white adipose tissue metabolism, eliciting distinct effects depending on the organism's feeding status.

- Yu X, Chen S, Funcke J-B, Straub LG, Pirro V, Emont MP, et al. The GIP receptor activates futile calcium cycling in white adipose tissue to increase energy expenditure and drive weight loss in mice. *Cell Metab*. 2025; 37:187-204.

- GIP acts on white adipocytes to promote energy expenditure through a mechanism that involves a futile calcium cycle.

- Valdecantos MP, Ruiz L, Folgueira C, Rada P, Gomez-Santos B, Solas M, et al. The dual GLP-1/glucagon receptor agonist G49 mimics bariatric surgery effects by inducing metabolic rewiring and inter-organ crosstalk. *Nat Commun*. 2024;15:10342

- Glucagon agonism, when added to GLP-1 agonism, enhances weight loss by inducing energy expenditure and promoting the browning of adipose tissue.

## Data Availability

No datasets were generated or analysed during the current study.
